# STALLION: a stacking-based ensemble learning framework for prokaryotic lysine acetylation site prediction

**DOI:** 10.1093/bib/bbab376

**Published:** 2021-09-17

**Authors:** Shaherin Basith, Gwang Lee, Balachandran Manavalan

**Affiliations:** Department of Physiology, Ajou University School of Medicine, Republic of Korea; Department of Molecular Science and Technology, Ajou University, Suwon 16499, Republic of Korea; Department of Physiology, Ajou University School of Medicine, Republic of Korea

**Keywords:** lysine acetylation sites, bioinformatics, stacking strategy, machine learning, feature optimization, performance assessment

## Abstract

Protein post-translational modification (PTM) is an important regulatory mechanism that plays a key role in both normal and disease states. Acetylation on lysine residues is one of the most potent PTMs owing to its critical role in cellular metabolism and regulatory processes. Identifying protein lysine acetylation (Kace) sites is a challenging task in bioinformatics. To date, several machine learning-based methods for the *in silico* identification of Kace sites have been developed. Of those, a few are prokaryotic species-specific. Despite their attractive advantages and performances, these methods have certain limitations. Therefore, this study proposes a novel predictor STALLION (STacking-based Predictor for ProkAryotic Lysine AcetyLatION), containing six prokaryotic species-specific models to identify Kace sites accurately. To extract crucial patterns around Kace sites, we employed 11 different encodings representing three different characteristics. Subsequently, a systematic and rigorous feature selection approach was employed to identify the optimal feature set independently for five tree-based ensemble algorithms and built their respective baseline model for each species. Finally, the predicted values from baseline models were utilized and trained with an appropriate classifier using the stacking strategy to develop STALLION. Comparative benchmarking experiments showed that STALLION significantly outperformed existing predictor on independent tests. To expedite direct accessibility to the STALLION models, a user-friendly online predictor was implemented, which is available at: http://thegleelab.org/STALLION.

## Introduction

The final step of the ‘central dogma’ of molecular biology is the translation process, where RNA codes for specific proteins [1]. Protein post-translational modifications (PTMs) are important owing to their implications in several biological processes, including cell cycle modulation, DNA repair, gene activation, gene regulation and signaling processes. PTMs are reversible or irreversible chemical changes that occur in the later stages of protein biosynthesis [2, 3]. PTMs can occur in a single amino acid residue or multiple residues resulting in changes in the chemical properties of altered sites [4]. Reversible modifications include covalent modifications, whereas irreversible changes include proteolytic modifications [5]. PTMs can affect several properties of proteins, such as cell–cell/cell–matrix interactions, enzyme assembly and function, molecular trafficking, protein–protein interactions (PPIs), protein folding, protein localization, protein solubility, protein lifespan and receptor activation, thus acting as an important regulatory tool in protein function [6, 7]. Over 400 different types of PTMs have been identified ranging from the addition of small chemical or complex groups (viz. acetylation, methylation, phosphorylation, etc.) to the addition of polypeptides (viz. ubiquitylation and SUMOylation; [8]). The lysine residue undergoes the largest number of PTMs with at least 15 PTM types [8]. According to the dbPTM database statistics, phosphorylation, acetylation and ubiquitination are the three major types that cover >90% of reported PTMs [9].

Lysine acetylation (Kace) is one of the most important ubiquitous PTMs and is highly conserved in both prokaryotes and eukaryotes [10]. This modification is a covalent PTM catalyzed by lysine acetyl transferases (KATs), where the acetyl group (CH_3_CO) from acetyl coenzyme A is transferred to either the free α-amino group (NH_3_^+^) of the N-terminal residue (Nα-acetylation) or to the ε-amino group of internal lysine (Nε-acetylation) at specific sites [11]. Acetylation is of three types, viz. Nα-, Nε- and O-acetylation. Nε- and O-acetylations are reversible modifications, whereas Nα-acetylation is an irreversible one [12]. Nα-acetylation is common in eukaryotes [13], whereas Nε-acetylation is more biologically substantial, playing prominent roles in actin nucleation, cell cycle regulation, chromatin stability, cell metabolism, nuclear transport and PPIs [14]. Dysregulation of Kace has also been linked to aging and several diseases including cancer, immune disorders and cardiovascular and neurological diseases [15, 16]. Given that acetylation is important in cell biology and disease pathologies, identifying Kace sites is necessary for understanding its modulatory mechanism.

Recently, several experimental methods, including radioactivity chemical methods, mass spectrometry and chromatin immunoprecipitation, have been developed to detect Kace PTM sites [17]. Owing to the latest innovations in science and technology, our ability to detect Kace sites has improved drastically; however, considering the proteome size, we have only uncovered a minute portion of the lysine ‘modifyome’. Moreover, testing every lysine residue in a single protein is laborious. The intricacies involved in the experimental identification of Kace sites (time-consuming, expensive, labor intensive and low throughput) have led to a plethora of computational approaches devised to predict potential candidates for experimental validation, particularly machine learning (ML) tools, which have become increasingly prevalent for their speedy and accurate predictions. In the last decade, several ML techniques have been developed to identify Kace sites in prokaryotes and eukaryotes [18–22].

Currently, more than a dozen Kace prediction tools are available, such as PAIL [23], LysAcet [24], EnsemblePail [25], N-Ace [26], BPBPHKA [27], PLMLA [28], PSKAcePred [29], KAcePred [31], LAceP [31], AceK [32], SSPKA [33], iPTM-mLys [34], KA-predictor [35], ProAcePred [36], ProAcePred 2.0 [37], Ning *et al*. [38] and DNNAce [39]. Most predictors were designed for identifying acetylation in eukaryotes and lacked species specificity. However, there are a few existing predictors that have been developed for identifying Kace in prokaryotes. SSPKA and KA-predictor were developed for both eukaryotic and prokaryotic acetylation site predictions, which included two prokaryotes, *Escherichia coli* and *Salmonella typhimurium*, thus underscoring the importance and necessity of a species-specific model. Chen *et al.* [36] developed a predictor called ProAcePred for nine prokaryotic species, *Archaea, Bacillus subtilis*, *Corynebacterium glutamicum*, *Erwinia amylovora*, *E. coli, Geobacillus kaustophilus, Mycobacterium tuberculosis, S. typhimurium* and *Vibrio parahemolvticus.* Later, the same group [36] developed the updated version of ProAcePred predictor called ProAcePred 2.0 [37] for six prokaryotic species: *B. subtilis*, *C. glutamicum*, *E. coli*, *G. kaustophilus, M. tuberculosis* and *S. typhimurium*. The training dataset was marginally larger than utilized in ProAcePred. Such ML studies provide an opportunity to understand differences in substrate site specificity between prokaryotic and eukaryotic species.

Although progress has been made in the computational prediction of Kace sites, a few limitations need to be addressed. First, most of the state-of-the-art approaches used simple ML algorithms such as support vector machine (SVM) or random forest (RF) to train the model. Owing to the advancement in cutting-edge technologies, advanced ML approaches, such as deep learning (DL) [40, 41], iterative feature representation [42] or ensemble-based stacking approach [43, 44], could be utilized for developing a more robust and stable predictor to enhance the predictive performance of Kace sites. Second, the feature space used by the existing methods in Kace prediction is rather limited. Finally, the state-of-the-art methods used simple feature selection technique to identify the optimal feature subset. Unfortunately, such simple approaches may overlook the critical features present in Kace site prediction.

Considering these limitations, we developed a novel stacking-based predictor known as STALLION (STacking-based Predictor for ProkAryotic Lysine AcetyLatION) to enhance the accurate prediction of Kace sites in six different prokaryotic species. Major advantages of our proposed method over other state-of-the-art methods could be summarized as follows: (i) STALLION is the first stacking ensemble-based predictor for the identification of Kace sites in prokaryotes; (ii) We comprehensively evaluated and compared 11 different encoding schemes for each species with an attempt to extract patterns representing a wide range of sequence, position-specific and physicochemical characteristics. Subsequently, we identified optimal feature set using three different computationally intensive approaches separately for five popular tree-based ensemble algorithms and trained the base classifiers and (iii) A stacked-model STALLION was trained with an appropriate classifier using the predicted information from the base classifiers and 5-fold cross-validation. Comparative analysis on independent datasets showed that the STALLION significantly outperformed existing predictor, thus highlighting the significance of utilizing our systematic approach in STALLION for Kace prediction.

## Materials and methods

### Training and independent datasets

Recently, Chen *et al.* [37] constructed novel nonredundant datasets, based on the PLMD database [45] (http://plmd.biocuckoo.org), for six species, *B. subtilis*, *C. glutamicum*, *E. coli*, *G. kaustophilus*, *M. tuberculosis* and *S. typhimurium*. Consequently, CD-HIT [46] was applied to eliminate the homologous sequences by setting the threshold of sequence identity to 30%, which is immensely valuable for avoiding overestimation during cross-validation or model training. While constructing the dataset, the authors experimented using different fragment sizes and identified the optimal size as 21-residue-long sequence segments with K at the center. The segments were defined as positive samples (Kace) if the central K residue acetylation was experimentally validated, otherwise they were deemed negative (non-Kace) samples. Notably, central K lacking residues or the gap at either terminus was replaced with a dummy atom ‘O’. Utilizing these datasets, they developed a species-specific Kace site predictor called ProAcePred 2.0.

We utilized the same dataset for the current study because they were recently constructed and used a rigorous approach to identify optimal length. In general, developing a prediction model using such a high-quality dataset may have more comprehensive practical applications [47]. A statistical summary of the training and independent datasets for each species is shown in Table 1. We employed balanced training datasets for prediction model development and imbalanced independent datasets to check the model robustness.

**Table 1:** A statistical summary of the training and independent datasets for six species

Species	Positive	Negative	Positive	Negative
*E. coli*	6592	6592	361	1384
*C. glutamicum*	1052	1052	83	830
*M. tuberculosis*	865	865	68	575
*B. subtillis*	1571	1571	125	1165
*S. typhimurium*	198	198	10	217
*G. kaustophilus*	206	206	17	192

*Note:* The first column represents the species, the second and third columns represent the positive and negative samples of the training dataset, and the fourth and fifth columns represent the positive and negative samples of the independent dataset.

### Selection of feature encoding schemes

To create an efficient ML-based method for Kace prediction, several different feature encoding schemes were employed to encode 21 types of amino acids [20 standard amino acids and dummy residues for gap (O)]. In total, we employed 11 encoding schemes that can be grouped into three major types: (i) sequence-based features include numerical representation of amino acid (NRA), binary encoding (BINA), amino acid composition (AAC), dipeptide composition (DPC) and conjoint triad (CTF); (ii) physicochemical properties based features include amino acid index (AAI), grouped dipeptide composition (GDPC), grouped tripeptide composition (GTPC)*,* Composition of k-Spaced Amino Acid Group Pairs (CKSAAGP) and Zscale and (iii) position-specific scoring matrices include BLOSUM62. A brief description of these 11 feature encoding schemes is as follows:

#### Sequence-based features

##### Numerical representation for amino acids features (NRF)

In NRF encoding, protein sequences are converted into numerical values [48] by mapping amino acids in an alphabetical order. The 21 amino acids are represented as 0.0–2.0 with an interval of 0.1. We ignored the central K residue from a given 21-residue segment and considered 10 upstream and 10 downstream sequences, resulting in a 20-dimensional (20D) feature vector.

##### Binary encoding (BINA)

In BINA encoding, each amino acid converts into a segment of a 21D orthogonal binary vector [49]. For example, alanine, cysteine and glutamic acid are represented as [1, 0, 0, 0, 0, 0, 0, 0, 0, 0, 0, 0, 0, 0, 0, 0, 0, 0, 0, 0, 0], [0, 1, 0, 0, 0, 0, 0, 0, 0, 0, 0, 0, 0, 0, 0, 0, 0, 0, 0, 0, 0] and [0, 0, 1, 0, 0, 0, 0, 0, 0, 0, 0, 0, 0, 0, 0, 0, 0, 0, 0, 0, 0], respectively. The dummy amino acid ‘O’ is represented as [0, 0, 0, 0, 0, 0, 0, 0, 0, 0, 0, 0, 0, 0, 0, 0, 0, 0, 0, 0, 1]. Thereafter, we obtained a 441D vector for the given sequence with a length of 21.

*Amino acid composition (AAC):* AAC computes the frequency of 21 amino acids from the given protein fragment sequence, which has been used as a descriptor for several peptide function predictions [50, 51]. The frequency of each amino acid is normalized into 0–1 by dividing the sequence length. AAC resulted in a 21D feature vector.

##### Di-peptide composition (DPC)

Twenty-one amino acids generate 441 (21 × 21) dipeptides. DPC computes the percentage of all possible dipeptide combinations from the given sequence, reflecting each protein sequence global information and local order amino acids information. Notably, DPC has been widely applied to capture segmental information around PTMs [52].

##### Conjoint triad features (CTF)

In CTF, 20 amino acids were categorized into seven classes ({VGA}, {PFLI}, {STMY}, {WQNH}, {RK}, {ED} and {C}) according to their volumes of side chains and dipoles [53]. Notably, we added a dummy atom to the first class {VGAO}. CTF considers the properties of one amino acid and its vicinal amino acids by regarding any three contiguous amino acids as a single unit. A 343D vector represents a given sequence.

#### Physicochemical properties-based features

##### Amino acid index (AAI)

AAI is the publicly available database that represents the physicochemical properties of amino acids as the most intuitive features for describing biochemical reactions (https://www.genome.jp/aaindex/; [54]). We utilized 531 physicochemical properties from the database as employed in the previous study [55] and encoded the given sequence.

##### Grouped di-peptide composition (GDPC)

Each amino acid has a specific physicochemical property. Accordingly, they have been categorized into five different groups: aliphatic (IMLVAG), aromatic (WYF), positively charged (HRK), negatively charged (ED) and uncharged (QNPCTSO). We assigned dummy residue (O) to the uncharged group. By utilizing these five groups, GDPC are classified into 25 classes that result in a 25D feature vector [56, 57].

##### Grouped tri-peptide composition (GTPC)

In GTPC [58, 59], the tri-peptide composition is classified into 125 classes by employing five categories as mentioned in GDPC, which result in a 125D vector.

##### Composition of k-spaced amino acid group pairs (CKSAAGP)

The CKSAAGP also calculates the frequency of amino acid pairs separated by *k* residues (the value of *k* is 0–5). Unlike the composition of *k*-spaced amino acid pairs [58], it classifies them into five categories based on the physicochemical properties of amino acids, and subsequently classifies the properties of the dipeptide compositions into 25 categories. Thus, the amino acid pair gives a total of 25 descriptors, and the number of divided residues is 0–5, such that a 150D vector is finally formed.

##### Z-scale (Zscale)

In Zscale, each amino acid is characterized by five physicochemical descriptor variables, according to Sandberg *et al.* [59]. A given sequence is converted into 105 (5 × 21) D vector by incorporating these five physicochemical descriptors.

#### Position-specific scoring matrices

##### BLOSUM62 (BLOS)

BLOSUM62 matrix is commonly applied in a BLAST sequence alignment program. Here, it was used to convert the protein sequence to describe the similarity of two sequence segments. Generally, this substitution matrix is applied to study sequence conservation of related proteins in large databases, which has been used as features in several predictors [60, 61]. Each row in the BLOSUM62 matrix can be used to encode one of the 20 amino acids. Therefore, we can encode according to the BLOSUM62 matrix, forming a feature vector of 420 (20 × 21) D.

### Selection of ML algorithms

In this study, we employed six different classifiers that included five decision-tree-based classifiers (RF [62], extreme gradient boosting algorithm (XGB) [63], AdaBoost (AB) [64], gradient boosting (GB) [65], extremely randomized tree (ERT) [66]) and SVM [67]. Generally, decision-tree-based algorithms can handle unnormalized features unlike other supervised and DL algorithms [68]. Hence, we only employed these five classifiers for the baseline model construction. However, six classifiers were used for meta-model construction and the appropriate one was selected. These classifiers have been widely applied in numerous successful applications in computational biology and bioinformatics [49, 69–74]. The detailed procedure regarding the implementation of each classifier is in line with our previous studies [75–78]. Generally, K-fold cross-validation analysis is required to train or develop the prediction model [79]. We employed 5-fold cross-validation and identified the optimal hyperparameters using a grid search approach. The grid search space for each classifier is provided in Supplementary Table S1.

### General framework of stallion

**Figure 1:**
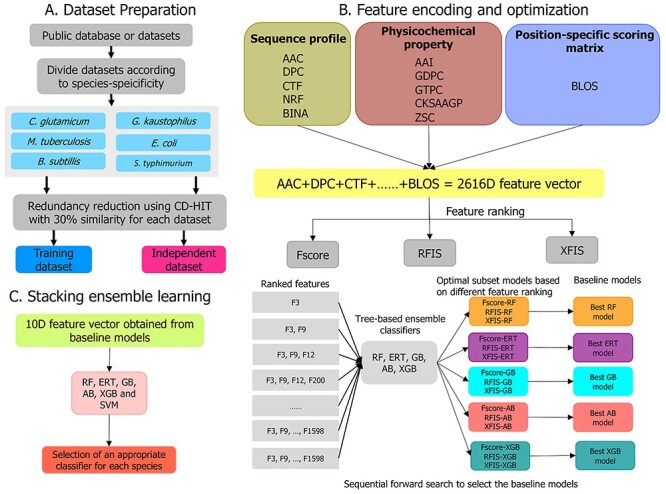
An overview of the STALLION framework for predicting prokaryotic lysine acetylation sites. Schematic display of the three stages in the construction of STALLION is shown.

A stacking ensemble learning-based framework of STALLION is summarized in Figure 1. It involves three crucial steps in the overall workflow and is described below:

#### Feature representation

The sequences in the training dataset of each species are encoded based on AAI (525D), AAC (21D), DPC (441D), CKSAAGP (150D), CTF (343D), Zscale (105D), BINA (441D), BLOS (420D), GTPC (125D), GDPC (25D) and NRF (20D) encoding schemes. We linearly integrated these 11 encodings for each sequence and obtained a 2616D feature vector. Thus, the training dataset of *B. subtilis*, *C. glutamicum*, *E. coli*, *G. kaustophilus*, *M. tuberculosis*, and *S. typhimurium* are represented as a 3142 × 2616, 2104 × 2616, 13,184 × 2616, 412 × 2616, 1730 × 2616 and 396 × 2616 matrix, respectively.

#### Feature optimization and selection

Each sequence contains a high-dimensional feature vector (2616D) that may include irrelevant or redundant information. Consequently, the predictive performance decreased and required vast computational resources during model training [80, 78]. We employed a two-step feature selection strategy to select the most informative features from the original feature dimension [80]. In the first step, each feature gets a score based on the scoring functions. Here, we employed three different scoring functions, viz. *F*-score, feature importance score (FIS) estimated by RF (RFIS) and FIS calculated by XGB (XFIS) according to their ability to distinguish Kace sites from non-Kace sites. Thereafter, we sorted the original feature dimension in descending order based on their scores. In total, we generated three feature lists (*F*-score, RFIS and XFIS), where *F*-score and RFIS contained the top 2000 features and XFIS included features that have only non-zero value (~500 features).

Second, a sequential forward search (SFS) was applied independently on three feature lists to identify suboptimum feature subsets. Letter *r* and *s* denoted the ranked feature list and suboptimum subset, respectively. In SFS, *k* (*k* = 5 for *F*-score and RFIS; *k* = 2 for XFIS) moved most informative features from *r* to *s*, which was inputted into five different classifiers independently and the performance evaluated by employing a 5-fold cross-validation in *s*. This process was repeated until *r* became empty. Ultimately, the feature subset for each classifier that achieved superior performance in terms of Mathews correlation coefficient (MCC) was considered an optimal set for each species. Generally, one of the scoring functions and a classifier was to be used to determine the optimal feature set [48, 76]. However, we applied a systematic approach for identifying the optimal feature set, although this procedure is computationally extensive. As we used three different ranked lists and five different classifiers, we obtained 15 models for each species.

#### Stacking ensemble learning

For each classifier, we selected the best model from three different suboptimum subset models. Consequently, we obtained five optimal baseline models for each species. Predicted probabilities and class labels received from baseline models were combined and considered as a new feature vector (10D). In general, the product of baseline models was trained with logistic regression while developing the final prediction model [48, 75]. However, we explored six classifiers that included the five tree-based classifiers and SVM. The reason for including SVM is that the new feature vector was in the range of 0–1 and can be handled well by SVM. All these classifiers were trained using ten randomized 5-fold cross-validation procedures. Given that MCC is our objective function during 5-fold cross-validation, it might be possible to overfit the prediction model to attain the highest MCC. Therefore, we repeated 5-fold cross-validation procedures ten times by randomly partitioning the training dataset, leading to 10 optimal feature sets for each classifier. For instance, SVM of C and $\gamma$ parameters have ten values each. However, we selected the median parameters of C and $\gamma$ for developing the final prediction model. Such randomized cross-validation techniques can avoid overfitting [47]. Finally, the average performances obtained from the randomized 5-fold cross-validations were compared for selecting the best model for each species.

### Additional feature encoding

We also tested K-nearest neighbor (KNN) encoding in this study, which is not part of STALLION. KNN encoding makes features for a given sequence based on the similarity of that sequence to the *n* samples from the training dataset (KAce and non-KAce). In particular, for a given two sequences *R_1_* and *R_2_* with the fixed length, the similarity score *F*(*R_1_*, *R_2_*) is computed as follows:

(1) \begin{equation*} F\left({R}_1,{R}_2\right)=1-\frac{\sum_{j=1}^K\mathrm{score}\left({R}_1(j),{R}_2(j)\right)}{K} \end{equation*}

where ${R}_1$ and ${R}_2$ represent amino acid residues of two sequences at the *j*th position, and *K* is the sequence length. For two amino acids *m* and *n*, the similarity score is defined as follows:

(2) \begin{equation*} Sim\left(m,n\right)=\frac{M\left(m,n\right)-\min (A)}{\max (A)-\min (A)} \end{equation*}

where (*m*, *n*) similarity score for two amino acids derived from the BLOSUM 62 substitution matrix [81], *A* is the substitution matrix and min(*A*)/max(*A*) represents the smallest/largest value in the matrix, respectively. In this study, we set *X* = 2, 4, 8, 16, 32, 64 and 128 to generate a 7D feature vector for a given sequence.

### Implementation

All cross-validations and independent evaluations were executed in a server with CentOS Linux 7.6 and Python 2.7.5. Notably, all ML classifiers (RF, ERT, GB, ERT and XGB v0.82; https://pypi.org/project/xgboost/) were built and optimized by Scikit-learn v0.18.1 package [82]. We computed three different (*F*-score, RFIS and XFIS) scoring functions to rank the features using the same package. In addition, feature encodings employed in this study were computed using our in-house code. Notably, a few open-source packages such as iLearn [56] and iFeature [57] can compute most feature encodings employed here.

### Performance evaluation strategies

Six performance measurements were applied to evaluate the model performance as widely employed in other studies [83, 84], including MCC, sensitivity (Sn), specificity (Sp), accuracy (ACC), balanced accuracy (BACC) and area under the receiver operating characteristics (ROC) curve (AUC). The definition of the metrics is as follows:

(3) \begin{equation*} \left\{\begin{array}{@{}c}\mathrm{Sn}=\frac{\mathrm{TP}}{\mathrm{TP}+\mathrm{FN}}\\{}\mathrm{Sp}=\frac{\mathrm{TN}}{\mathrm{TN}+\mathrm{FP}}\\{}\mathrm{ACC}=\frac{\mathrm{TP}+\mathrm{TN}}{\mathrm{TP}+\mathrm{TN}+\mathrm{FN}+\mathrm{FP}}\\{} BACC= Sn\times 0.5+ Sp\times 0.5\\{}\mathrm{MCC}=\frac{\mathrm{TP}\times \mathrm{TN}-\mathrm{FP}\times \mathrm{FN}}{\sqrt{\left(\mathrm{TP}+\mathrm{FN}\right)\left(\mathrm{TP}+\mathrm{FP}\right)\left(\mathrm{TN}+\mathrm{FP}\right)\left(\mathrm{TN}+\mathrm{FN}\right)}}\end{array}\right. \end{equation*}

where TP, TN, FP and FN, respectively denote true positives, true negatives, false positives and false negatives. Furthermore, ROC curves and AUC values were used to assess overall performance.

## Results and discussion

### Performance evaluation between different feature encoding methods and classifiers

**Figure 2:**
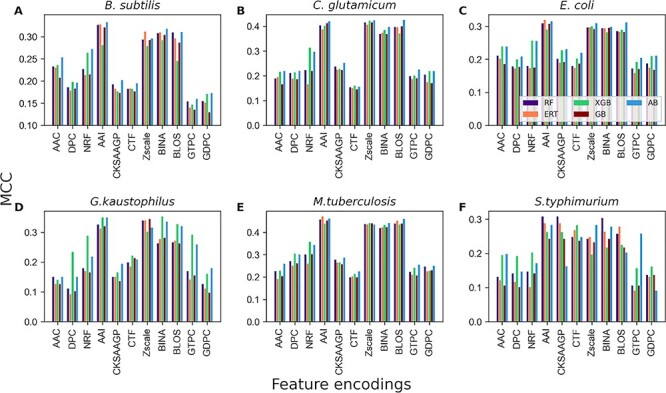
Performances of five different ML classifiers in distinguishing between Kace and non-Kace with respect to 11 feature descriptors. (**A**) *B. subtilis* (**B**) *C. glutamicum*, (**C**) *E. coli*, (**D**) *G. kaustophilus*, (**E**) *M. tuberculosis* and (**F**) *S. typhimurium*.

We systematically investigated the effect of various feature encodings and classifiers in prokaryotic Kace site predictions by employing five tree-based ensemble classifiers (RF, GB, ERT, XGB and AB) and eleven feature encodings including sequence-based, physicochemical properties and position-specific scoring matrix. We performed a ten times randomized 5-fold cross-validation test for constructing each model for each species dataset and compared the performances among 55 models (11 encodings × 5 classifiers). Figure 2 shows that four encodings (AAI, Zscale, BINA and BLOS) achieved similar performances and were significantly better than the other seven encodings for most prokaryotic species (*B. subtilis*, *C. glutamicum*, *E. coli*, *G. kaustophilus* and *M. tuberculosis*). However, we noted that six encodings achieved similar performances and were significantly higher than the other five encodings (AAC, DPC, NRF, GTPC and GDPC) for *S. typhimurium*. Overall, four encodings (AAI, Zscale, BINA and BLOS) were found to be superior compared to their counterparts. Nevertheless, other encodings also possessed essential information to support Kace site prediction. To get an overview of the performance of each classifier on Kace prediction, we computed an average performance of 66 models (11 encodings × 6 species) for each classifier. The results showed that AB, XGB, RF, ERT and GB achieved average MCCs of 0.261, 0.255, 0.241, 0.232 and 0.230, respectively. Notably, all classifiers performed reasonably well in Kace site prediction; however, AB was found to be marginally superior. Rather than searching for the best model, integrating the above information and developing a robust model is admissible. In this study, we applied a stacking approach similar to recent studies [76, 85, 86].

### Identification of the optimal model of each classifier for six species

As mentioned in the methods section, we applied three different scoring functions to rank features, each with its own pros and cons. For example, *F*-score and RFIS assign a relative score for all given features. However, XFIS excludes ~70% of the features and designates a relative score for the remaining features. Supplementary Figure S1 shows the performances of the five classifiers for different feature sets in the *C. glutamicum* species. Here, we observed that the performance increased steadily, achieved maximum accuracy and subsequently remained in an equilibrium state for most classifiers based on the *F*-score (Supplementary Figure S1A) and RFIS (Supplementary Figure S1B). However, for XFIS, performance increased slowly until the optimal one and subsequently deteriorated while adding more features (SupplementaryFigure S1C), regardless of the classifiers.

The size of the optimal feature set varied among five classifiers for each one of the three different approaches (*F*-score, RFIS and XFIS). For example, RF, ERT, GB, XGB and AB possessed 1000, 520, 790, 260 and 410 optimal feature sets, respectively, from *F*-score identified by SFS. The corresponding classifiers had 140, 1290, 211, 120 and 150D optimal feature sets from RFIS and 30, 38, 31, 52 and 44D optimal feature sets from XFIS. Similarly, the best model for each classifier from three different approaches showed different sizes of optimal feature sets. For example, RF possessed three models with 1000, 140 and 40D optimal feature sets. However, we selected the best model based on maximal accuracy. The same procedure was followed for the other species and the best three models were selected for each classifier, whose performances were compared with the control.

**Figure 3:**
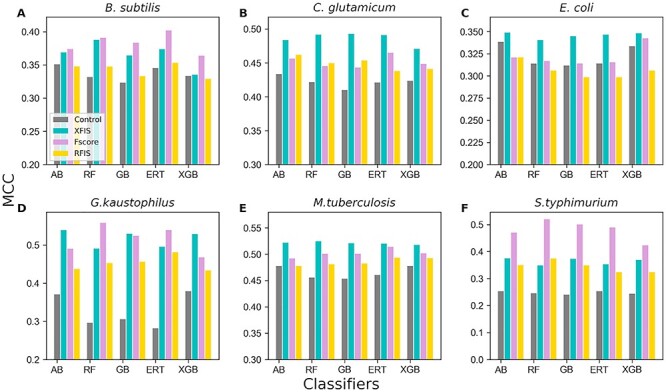
Performance comparisons between the control and the three optimal models for each classifier. Sequential forward search identified the optimal model for each classifier from Fscore, RFIS, and XFIS. (**A**) *B. subtilis* (**B**) *C. glutamicum*, (**C**) *E. coli*, (**D**) *G. kaustophilus*, (**E**) *M. tuberculosis* and (**F**) *S. typhimurium*.

Figure 3 shows that the performances of the optimal model were consistently better than the control, thus indicating the necessity of feature selection techniques to exclude irrelevant information from the original dimension. For three species (*C. glutamicum*, *E. coli* and *M. tuberculosis*), the optimal feature sets obtained from XFIS achieved superior performances for five classifiers compared to their counterparts (*F*-score and RFIS). In two species (*S. typhimurium* and *B. subtilis*), the optimal feature set extracted from *F*-score achieved excellent performances for five classifiers compared to their counterparts (XFIS and RFIS). However, for *G. kaustophilus*, the optimal feature sets derived from the *F*-score showed improved performance for RF and ERT classifiers. The remaining three classifiers showed improved performance upon the acquisition of optimal features from XFIS. Unexpectedly, the optimal feature set derived from RFIS did not show the best performance. Notably, the best models for five classifiers have been considered as baseline models in each species and utilized for subsequent analysis. Overall, our systematic feature selection analysis suggests that it is essential to apply different scoring functions to rank features and employ different classifiers individually for SFS to obtain their corresponding optimal feature set.

### Construction of STALLION

**Figure 4:**
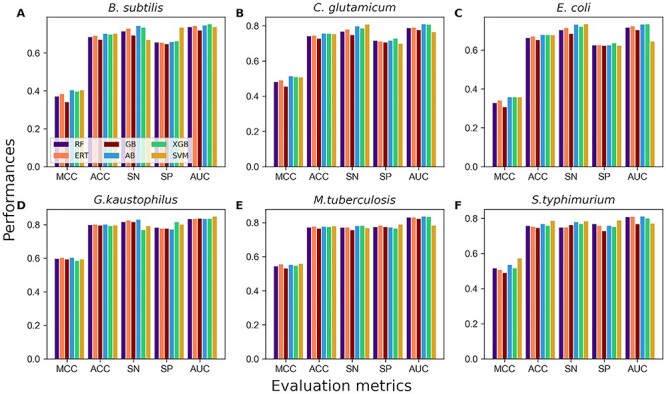
Performance comparison of six different classifiers for predicting Kace sites during stacking strategy and 10 randomized 5-fold cross-validation. Performances expressed in terms of MCC, ACC, Sn, Sp and AUC. (**A**) *B. subtilis* (**B**) *C. glutamicum*, (**C**) *E. coli*, (**D**) *G. kaustophilus*, (**E**) *M. tuberculosis* and (**F**) *S. typhimurium*.

Stacking is an ensemble technique that considers different predictive models to generate a stable stacked model. This approach employs an efficient scheme to decrease the generalization error rate of various predictive models [87–89]. The predicted values (predicted probability of Kace and class label) from the five baseline models were combined to generate a 10D feature vector. Unlike previous approaches [44, 76], we systematically evaluated six different classifiers by training with a new 10D feature vector using 10 randomized 5-fold cross-validations (Figure 4). The results showed that the five classifiers (RF, ERT, AB, XGB and SVM) achieved similar performances, which were marginally better than GB. Among these five classifiers, we selected the AB classifier for three species (*B. subtilis*, *C. glutamicum* and *G. kaustophilus*), the SVM classifier for two species (*M. tuberculosis* and *S. typhimurium*), and the XGB classifier for *E. coli*, whose performances are marginally superior to its counterparts. Six species models were commonly named as STALLION that achieved ACC, MCC and AUC of 0.403, 0.700 and 0.745, respectively for *B. subtilis*; 0.513, 0.756 and 0.809, respectively for *C. glutamicum*; 0.357, 0.678 and 0.733, respectively for *E. coli*; 0.603, 0.801 and 0.836, respectively for *G. kaustophilus*; 0.557, 0.779 and 0.782, respectively for *M. tuberculosis*; and 0.571, 0.785 and 0.770, respectively for *S. typhimurium*.

### Comparison of STALLION with single feature-based models

**Figure 5:**
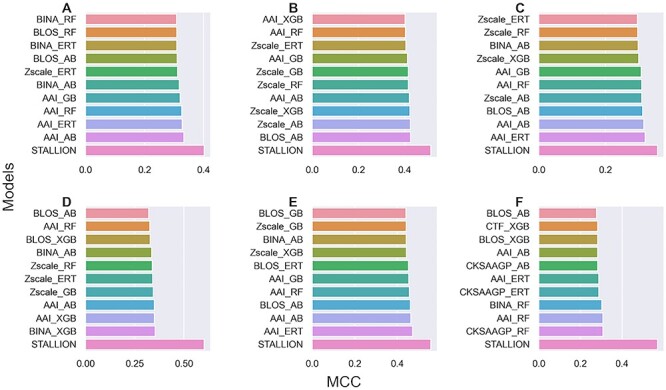
Performance comparison between STALLION and single feature-based models in classifying Kace from non-Kace sites during cross-validation. (**A**) *B. subtilis* (**B**) *C. glutamicum*, (**C**) *E. coli*, (**D**) *G. kaustophilus*, (**E**) *M. tuberculosis* and (**F**) *S. typhimurium*.

To show the advantage of our proposed stacked approach, we compared STALLION with the single feature-based models. We selected the top 10 single feature-based models from Figure 2 and compared them with STALLION for six species. Figure 5 shows that STALLION significantly outperformed single feature-based models for all six species, whose MCC was 6.9–9.4% higher for *B. subtilis*, 8.8–11.1% higher for *C. glutamicum*, 3.7–6.1% higher for *E. coli*, 24.9–28.2% higher for *G. kaustophilus*, 8.6–11.7% higher for *M. tuberculosis* and 26.2–29.3% higher for *S. typhimurium*. The superior performance of STALLION over the single feature-based models was primarily due to the novelty introduced in our approach, which included (i) feature fusion strategy, (ii) selecting the optimal feature set from hybrid features for each classifier independently and their respective baseline model construction and (iii) selecting an appropriate classifier for stacking model construction.

**Figure 6:**
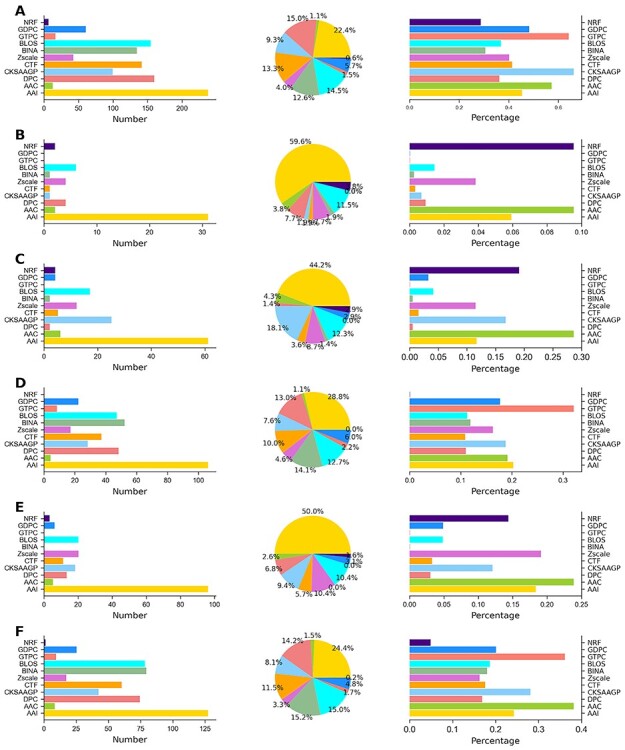
Distribution analysis of generated optimal feature sets across the six species. Panels (**A**)–(**F**) illustrate distributions of feature types included in optimal feature sets for *B. subtilis*, *C. glutamicum*, *E. coli*, *G. kaustophilus*, *M. tuberculosis* and *S. typhimurium*, respectively. Each panel containing three figures represent number of each feature type selected in the optimal feature set, portion of the types of features selected in the optimal feature set, and percentage of each feature type selected in the optimal feature set.

### Feature contribution analysis

To understand the contribution of different features in the optimal feature set for each species, we analyzed their composition and distribution. It is worth mentioning that five classifier models have different optimal feature subsets for each species. Instead of focusing on each subset, we considered the maximum size of the optimal feature subset that potentially includes the other four subsets for five species (*B. subtilis*, *C. glutamicum*, *E. coli*, *M. tuberculosis* and *S. typhimurium*). For example, in *C. glutamicum*, RF, ERT, GB, XGB and AB contained 30, 38, 31, 52 and 44D optimal feature subsets, respectively. Here, 52D had other feature subsets. However, in *G. kaustophilus*, different optimal subsets were combined to investigate their role.

**Figure 7:**
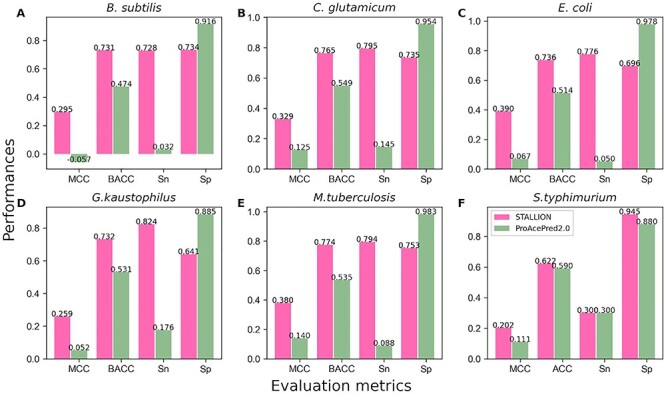
Performance comparison between STALLION and ProAcePred2.0 in classifying Kace from non-Kace sites during independent test. (**A**) *B. subtilis* (**B**) *C. glutamicum*, (**C**) *E. coli*, (**D**) *G. kaustophilus*, (**E**) *M. tuberculosis* and (**F**) *S. typhimurium*.

Figure 6 indicates that the feature distribution in the optimal feature set among six species showed significant differences; however, some subtle similarities were noted. Particularly, AAI contributed 22.4%, 59.6%, 44.2%, 28.8%, 50.0% and 24.4% of the total optimal features for *B. subtilis*, *C. glutamicum*, *E. coli*, *G. kaustophilus, M. tuberculosis* and *S. typhimurium*, respectively. This result implies that the AAI feature contribution is important for six species, suggesting their critical importance in Kace prediction. Six encodings (AAC, DPC, CKSAAGP, CTF, Zscale and BLOS) consistently contributed to the optimal feature set for all species. Still, the contribution level varied among them suggesting a supporting role played in Kace prediction. Furthermore, we observed that GTPC and GDPC, GTPC, NRF and GTPC and BINA did not contribute to the final prediction for *C. glutamicum*, *E. coli*, *G. kaustophilus* and *M. tuberculosis*, respectively. Overall, apart from AAI, the rest of the feature contribution varied considerably among species, thus suggesting that Kace sites in these species might have different characteristics.

### Performance validation using the independent test

We further evaluated STALLION using independent datasets and compared their performances with the existing method. Since 2009, several computational tools have been reported for Kace site prediction. Notably, Chen *et al.* [37] recently evaluated species-specific ProAcePred 2.0 predictor using an independent dataset and compared the performance with existing methods, including species-specific ProAcePred, general predictors viz. EnsemblePail, PSKAcePred, Phosida and PLMLA. The result showed that ProAcePred 2.0 significantly outperformed generic predictors and their previous version ProAcePred. Therefore, only ProAcePred 2.0 was considered in this study for comparison and other methods were excluded for the following reasons: (i) comparing species-specific predictor with a generic predictor is unfair, which is evident from previous studies [36, 37] and (ii) ProAcePred 2.0 is the upgraded version of ProAcePred.

It is worth mentioning that the independent dataset for each species was submitted to the ProAcePred 2.0 web server (http://computbiol.ncu.edu.cn/PAPred) and the predictions were computed based on the given default threshold. Notably, the ProAcePred 2.0 returns Kace site and its predicted probability value, but not non-Kace predicted probability values. Therefore, it might be unfeasible to compute the AUC value with partial probability information. However, we compared the performances between two methods in terms of MCC, which is an intuitive and straightforward metric while dealing with an imbalanced dataset, as mentioned in [90]. Our evaluation results showed that STALLION achieved MCC of 0.295, 0.329, 0.390, 0.259, 0.380 and 0.202 for *B*. *subtilis*, *C*. *glutamicum*, *E*. *coli*, *G. kaustophilus*, *M. tuberculosis* and *S. typhimurium*, respectively (Figure 7 and Supplementary Table S2). STALLION outperformed ProAcePred 2.0 by >20.0% in MCC value for five species (*B*. *subtilis*, *C*. *glutamicum*, *E*. *coli*, *G. kaustophilus* and *M. tuberculosis*) and 9.1% in MCC value for *S. typhimurium.* STALLION provided better performance than ProAcePred2.0 because of the following: (i) Unlike ProAcePred 2.0, we excluded KNN feature encoding from the stacking framework based on our systematic analysis that identified the overfitting nature of KNN encoding during cross-validation (see section below); (ii) Unlike ProAcePred 2.0 simple feature selection approach, we employed a rigorous process by utilizing three different scoring functions and SFS to identify the optimal feature set independently for each classifier, which is time-consuming and (iii) Unlike a single model in ProAcePred 2.0, our stacking strategy integrates five tree-based ensemble baseline models leading to more accurate Kace site predictions.

**Figure 8:**
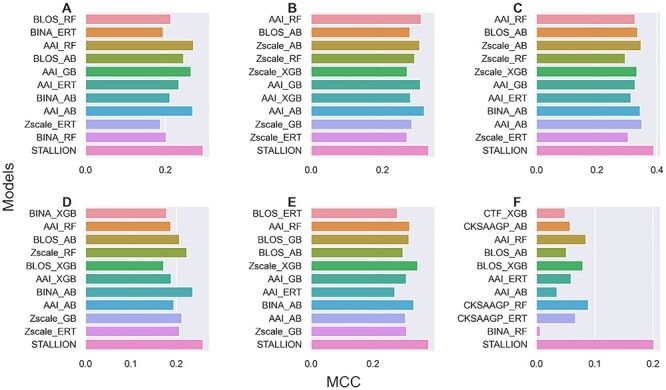
Performance comparison between STALLION and single feature-based models in classifying Kace from non-Kace sites during independent test. (**A**) *B. subtilis* (**B**) *C. glutamicum*, (**C**) *E. coli*, (**D**) *G. kaustophilus*, (**E**) *M. tuberculosis* and (**F**) *S. typhimurium*.

Like STALLION and the best single feature-based models’ cross-validation performance comparison, we carried out independent tests. Figure 8 shows that STALLION outperformed single feature-based models for all six species, whose MCC was 2.39–10.68% higher for *B. subtilis*, 1.18–6.08% higher for *C. glutamicum*, 4.0–9.5% higher for *E. coli*, 2.5–8.7% higher for *G. Kaustophilus*, 3.51–10.89% higher for *M. tuberculosis* and 11.29–19.54% higher for *S. typhimurium*. These results yet again highlight the significance of our systematic approach in model construction.

### Overfitting nature of KNN encoding in Kace prediction

KNN feature encoding is widely applied for the identification of PTM sites, including previous Kace site prediction methods [36, 37]. Similar to previous studies, we also incorporated it into 11 encodings in the stacking framework. The preliminary result showed that all species models’ prediction performance significantly improved compared with the STALLION during cross-validation. However, the corresponding model performance with independent datasets was slightly better than the random prediction and considerably lower than the STALLION. Hence, we excluded KNN encoding from the stacking framework (STALLION). To better understand the phenomenon, we developed KNN-based five tree-based models for each species and examined their cross-validation and independent validation performances (Table 2). The results showed that four (RF, ERT, AB and XGB) out of five classifiers achieved similar performances, which were marginally better than GB with average AUCs of 0.895, 0.901, 0.888, 0.888, 0.895 and 0.872 for five models, namely *B. subtilis*, *C. glutamicum*, *E. coli*, *G. kaustophilus, M. tuberculosis* and *S. typhimurium*, respectively. The corresponding metrics on independent tests were 0.602, 0.665, 0.621, 0.597, 0.670 and 0.619 for the six species. The performance gap (difference in AUC) between the training and independent datasets in all six species significantly increased from 22.46 to 29.32%, clearly indicating the overestimation of KNN encoding during training regardless of the classifiers. Owing to the over-fitting nature of the KNN encoding scheme, we highly recommend testing KNN encoding transferability before incorporating it into any computational frameworks requiring huge computations.

**Table 2:** Performance comparison of various classifiers on KNN encoding during training and independent tests

Species	Classifier	MCC	ACC	Sn	Sp	AUC	MCC	ACC	Sn	Sp	AUC
*B. subtillis*	RF	0.599	0.799	0.757	0.840	0.907	0.105	0.573	0.608	0.569	0.630
	ERT	0.605	0.802	0.833	0.771	0.903	0.106	0.568	0.616	0.563	0.612
	GB	0.583	0.791	0.778	0.805	0.857	0.108	0.558	0.632	0.550	0.562
	AB	0.623	0.805	0.701	0.908	0.908	0.106	0.650	0.504	0.666	0.623
	XGB	0.652	0.799	0.597	1.000	0.899	0.070	0.702	0.368	0.737	0.581
*C. glutamicum*	RF	0.616	0.808	0.816	0.800	0.915	0.168	0.610	0.687	0.602	0.690
	ERT	0.599	0.799	0.786	0.813	0.917	0.168	0.610	0.687	0.602	0.676
	GB	0.590	0.795	0.806	0.784	0.828	0.124	0.590	0.627	0.587	0.608
	AB	0.646	0.823	0.844	0.801	0.922	0.203	0.607	0.759	0.592	0.678
	XGB	0.648	0.821	0.886	0.757	0.923	0.196	0.578	0.783	0.558	0.673
*E. coli*	RF	0.582	0.791	0.776	0.806	0.905	0.152	0.559	0.654	0.534	0.631
	ERT	0.578	0.789	0.806	0.772	0.905	0.151	0.559	0.651	0.535	0.624
	GB	0.576	0.788	0.780	0.797	0.824	0.131	0.543	0.645	0.517	0.582
	AB	0.610	0.804	0.755	0.852	0.909	0.241	0.622	0.693	0.604	0.657
	XGB	0.627	0.782	0.565	1.000	0.895	0.146	0.675	0.424	0.741	0.610
*G. kaustophilus*	RF	0.612	0.806	0.796	0.816	0.908	0.104	0.502	0.706	0.484	0.630
	ERT	0.631	0.816	0.811	0.820	0.912	0.061	0.479	0.647	0.464	0.625
	GB	0.626	0.813	0.801	0.825	0.835	0.058	0.474	0.647	0.458	0.582
	AB	0.646	0.823	0.816	0.830	0.866	0.011	0.445	0.588	0.432	0.555
	XGB	0.631	0.816	0.820	0.811	0.919	0.072	0.498	0.647	0.484	0.593
*M. tuberculosis*	RF	0.663	0.831	0.820	0.843	0.928	0.241	0.646	0.735	0.634	0.681
	ERT	0.652	0.826	0.836	0.816	0.917	0.237	0.641	0.735	0.628	0.666
	GB	0.636	0.818	0.809	0.827	0.853	0.206	0.612	0.721	0.597	0.667
	AB	0.685	0.842	0.806	0.878	0.932	0.234	0.656	0.706	0.650	0.689
	XGB	0.694	0.845	0.791	0.899	0.845	0.197	0.651	0.647	0.652	0.649
*S. typhimurium*	RF	0.540	0.770	0.768	0.773	0.893	0.096	0.542	0.700	0.535	0.615
	ERT	0.551	0.775	0.783	0.768	0.843	0.106	0.564	0.700	0.558	0.645
	GB	0.551	0.775	0.768	0.783	0.813	0.095	0.537	0.700	0.530	0.615
	AB	0.591	0.796	0.788	0.803	0.906	0.048	0.520	0.600	0.516	0.619
	XGB	0.602	0.801	0.823	0.778	0.907	0.003	0.507	0.500	0.507	0.603

*Note:* The first and the second columns represent the species and the ML classifiers. Columns 3–7 represent MCC, ACC, Sn, Sp and AUC of the ML classifier performance on the training dataset. Columns 8–12 represent MCC, ACC, Sn, Sp and AUC of the ML classifier performance on the independent dataset.

### Availability of online webserver

Publicly accessible web servers can help experimental or biomedical researchers to identify the putative functional sites, which will aid further experimental characterization. To help the user identify high-throughput putative Kace sites from six prokaryotic species, we implemented the STALLION web server, which is freely accessible at: http://thegleelab.org/STALLION. The STALLION web server is maintained by an Apache HTTP server and configured in a 16-core CentOs Linux 7.6 server machine with 64GB RAM and a 2 TB hard disk. We have given the detailed instructions for using the STALLION in the following link: http://thegleelab.org/STALLION/Staltutorial.html. In addition, we provided the server running time of our independent datasets in the above link.

## Conclusions

This study presented STALLION, a stacking framework for the accurate Kace site prediction from six different prokaryotic species. STALLION employed 11 distinct feature encoding schemes (categorized into three groups) to encode protein fragments. Subsequently, a rigorous feature selection approach was employed to carefully select the optimal feature set for each of the five different tree-based ensemble algorithms and constructed their respective baseline models for each species. Finally, the predicted output was derived from five baseline models which were trained with an appropriate classifier to build the stable, stacked STALLION models. Our proposed method STALLION outperformed the current state-of-the-art predictor for identifying Kace sites on the independent data sets across six different species. It is expected that STALLION methodology and a user-friendly web server based on the stacked model for six prokaryotic species will expedite the discovery of putative Kace sites and greatly assist the effort of a broader research community for functional characterization. Our study identified that heterogeneous and complementary features derived from different perspectives helped to improve predictor performance. We will continually attempt to investigate other informative features, examine their contribution and refine our prediction platform.

Overall, the STALLION method has achieved a robust performance in Kace site prediction, whose prediction performance requires further improvement in several aspects. Novel computational frameworks have been reported recently, including a DL-based hybrid framework [86] and DL-based approaches with automatically generated features [91, 92]. In future, we will examine the possibility of these approaches and select the appropriate one to further improve prediction performance of Kace sites.

**Key Points**

- We propose a stacking framework STALLION and implement it as a user-friendly webserver for accurate identification of prokaryotic Kace sites.
- STALLION utilized 11 different features encoding schemes and combined five tree-based ensemble algorithms to build stable stacked models.
- Extensive benchmarking experiments demonstrated that STALLION outperformed its constituent baseline models in both training and independent datasets, thus highlighting its excellent generalization capability.

## Supplementary Material

Supplementary_bbab376

## Data Availability

Training and independent datasets used in this study could be freely downloaded by clicking the below link: http://thegleelab.org/STALLION/StalData.html

## Notes

**Shaherin Basith** is a research assistant professor in the Department of Physiology, Ajou University School of Medicine, Republic of Korea. Her main area of research is exploring the structure–function relationships of proteins using state-of-the-art molecular modeling tools, phylogenetic analysis and biomolecular simulations. She is also actively involved in the application of machine learning tools for peptide and small molecule drug discovery.

**Gwang Lee** is a professor in the Department of Physiology, Ajou University School of Medicine, Republic of Korea. His main research interests include the integration of triple omics (transcriptomics, metabolomics and proteomics) for nanotoxicity studies, machine learning, neurodegenerative diseases and biomolecular simulations targeting therapeutically important proteins or enzymes.

**Balachandran Manavalan** is an assistant professor in the Department of Physiology, Ajou University School of Medicine, Republic of Korea. He is also an associate member of Korea Institute for Advanced Study, Republic of Korea. His research interests include artificial intelligence, bioinformatics, machine learning, big data and functional genomics.
