# Supplementary material for: STALLION: a stacking-based ensemble learning framework for prokaryotic lysine acetylation site prediction
Source: Brief Bioinform. 2021 Sep 17;23(1):bbab376. doi: 10.1093/bib/bbab376 (PMC8769686; doi:10.1093/bib/bbab376)
Supplement: Supplementary_bbab376 [file supplementary_bbab376.docx]

**STALLION: A Stacking-based Ensemble Learning Framework for Prokaryotic Lysine Acetylation Site Prediction**

Shaherin Basith^1^, Gwang Lee^1,2,^* and Balachandran Manavalan^1,^*

^1^Department of Physiology, Ajou University School of Medicine, Suwon 16499, Republic of Korea.

^2^Department of Molecular Science and Technology, Ajou University, Suwon, 16499, Republic of Korea

*Corresponding authors

Gwang Lee: [glee@ajou.ac.kr](mailto:glee@ajou.ac.kr)

Balachandran Manavalan: [bala@ajou.ac.kr](mailto:bala@ajou.ac.kr)

Table S1. Hyperparameters search range for six machine-learning classifiers.

| **Method** | **Parameter** | **Tested values** |
| --- | --- | --- |
| RF, ERT and GB | Number of estimators (n_estimators^[a]^) | 50–10000 with an interval of 25 |
|  | Maximum number of features considered per split (max_features^[a]^) | 1–10 with an interval of 1 |
|  | Minimum number of samples required to split an internal node (min_samples_split^[a]^) | 2–13 with an interval of 1 |
| AB | Number of estimators (n_estimators^[a]^)  maximum_depth  lerarning_rate | 20–500 with an interval of 20  1–10 with an interval of 1  [0.0001, 0.001, 0.01, 0.05, 0.1, 0.2, 0.25, 0.3, 0.5, 1.0] |
|  |  |  |
| XGB | n_estimator  lerarning_rate  maximum_depth  regularization parameter ($\varepsilon$) | 40–1000 with an interval of 20  [0.0001, 0.001, 0.01, 0.05, 0.1, 0.2, 0.25, 0.3, 0.5, 1.0]  2–10 with an interval of 2.  [0.0001, 0.001, 0.002, 0.01, 0.02, 0.05, 1.0] |
| SVM | Regularization parameter (C)^[a]^  RBF Kernel coefficient ($\gamma$)^[a]^ | np.logspace(-5, 10,base=2)  np.logspace(-10, 4,base=2) |

Column 1 represents the ML algorithms. Columns 2 and 3 respectively represent the parameter and the tested values. ^[a]^ Parameter name in the scikit-learn implementation.

**Table S2**. Performance comparison between STALLION and the best existing predictor on independent datasets.

| Species | Method | MCC | BACC | Sn | Sp | TP | TN | FP | FN |
| --- | --- | --- | --- | --- | --- | --- | --- | --- | --- |
| *B. subtillis* | STALLION | 0.295 | 0.731 | 0.728 | 0.734 | 91 | 855 | 310 | 34 |
|  | ProAcePred2.0 | -0.057 | 0.474 | 0.032 | 0.916 | 4 | 1067 | 98 | 121 |
| *C. glutamicum* | STALLION | 0.329 | 0.765 | 0.795 | 0.735 | 66 | 610 | 220 | 17 |
|  | ProAcePred2.0 | 0.125 | 0.549 | 0.145 | 0.954 | 12 | 792 | 38 | 71 |
| *E*. *coli* | STALLION | 0.390 | 0.736 | 0.776 | 0.696 | 280 | 963 | 421 | 81 |
|  | ProAcePred2.0 | 0.067 | 0.514 | 0.050 | 0.978 | 18 | 1353 | 31 | 343 |
| *G. kaustophilus* | STALLION | 0.259 | 0.732 | 0.824 | 0.641 | 14 | 123 | 69 | 3 |
|  | ProAcePred2.0 | 0.052 | 0.531 | 0.176 | 0.885 | 3 | 170 | 22 | 14 |
| *M. tuberculosis* | STALLION | 0.380 | 0.774 | 0.794 | 0.753 | 54 | 387 | 127 | 14 |
|  | ProAcePred2.0 | 0.140 | 0.535 | 0.088 | 0.983 | 6 | 566 | 10 | 62 |
| *S. typhimurium* | STALLION | 0.202 | 0.622 | 0.300 | 0.945 | 3 | 205 | 12 | 7 |
|  | ProAcePred2.0 | 0.111 | 0.590 | 0.300 | 0.880 | 3 | 191 | 26 | 7 |

**Figure S1.** A plot showing the sequential forward search procedure optimized by F-score (A), RFIS (B) and XFIS (C) for identifying Kace sites. The optimal feature set that peaks of ACC values marked by green filled circle during 5-fold cross-validation.
